# Modeling graphene oxide decorated with FeO, SO and NO

**DOI:** 10.1038/s41598-025-18685-5

**Published:** 2025-09-15

**Authors:** Hesham El Meligy, Khaled S. Amin, Hanan Elhaes, Medhat A. Ibrahim

**Affiliations:** 1 Electron Microscope & Thin Films Department, National Research Centre, 33 El-Bohouth St., 12622 Dokki, Giza Egypt; 2 Physics Department, Faculty of Science, Al-Azhar University, Cairo, Egypt; 3 Physics Department, Faculty of Women for Arts, Science and Education, Ain Shams University, 11757 Cairo, Egypt; 4 Spectroscopy Department, National Research Centre, 33 El-Bohouth St., 12622 Dokki, Giza Egypt; 5 Molecular Modeling and Spectroscopy Laboratory, Centre of Excellence for Advanced Science, National Research Centre, 33 El-Bohouth St., 12622 Dokki, Giza Egypt

**Keywords:** GrO, Decoration, DFT, B3LYP/ LANL2MB, DOS, Global reactivity descriptors and QTAIM, Chemistry, Materials science, Nanoscience and technology, Physics

## Abstract

In this computational investigation, the effect of decoration of graphene oxide (GrO) with three different species FeO, SO, and NO modulates its electronic structure and reactivity for potential electrode and sensing applications. All model structures (pristine graphene, GrO, GrO/FeO, GrO/SO, and GrO/NO) were optimized at the B3LYP/LANL2MB level of theory. We analyzed total dipole moments (TDM), HOMO/LUMO energy gaps (ΔE), global reactivity descriptors (I, A, µ, η, S, ω), density of states (DOS and PDOS), molecular electrostatic potential (MESP), Quantum Theory of Atoms in Molecules (QTAIM) topologies, and noncovalent interaction (NCI) patterns. Oxidation from Gr to GrO created a modest dipole moment (3.06 Debye) and reduced ΔE from 4.483 eV to 3.226 eV. Decoration with FeO raised the TDM to 14.26 Debye and decreased ΔE to 1.625 eV, while SO decoration yielded the largest TDM (20.38 Debye) and the smallest gap (0.576 eV). In contrast, NO decoration produced intermediate values (TDM = 2.90 Debye, ΔE = 2.412 eV). Global reactivity descriptors confirm that GrO/FeO and GrO/SO acquire strong electrophilic character and high softness, and GrO/NO retains moderate reactivity. DOS/PDOS analysis shows that Fe, S, and N introduce new states near the Fermi level, facilitating charge transfer. MESP maps identify electron-rich and -poor regions at functional sites, while QTAIM indicates a covalent Fe–O bond in GrO/FeO and hydrogen-bonding interactions in GrO/SO and GrO/NO. NCI analysis further supports the presence of van der Waals interactions at the decoration interfaces. Taken together, our results demonstrate that choice of decorating species enables precise tuning of GrO’s electronic and reactive properties, highlighting GrO/FeO and especially GrO/SO as promising candidates for enhanced electrode performance and gas sensing.

## Introduction

Graphene oxide which is abbreviated as GrO is a class of 2D carbon-based materials, it could be described also as is a single-layer sheet of graphite oxide^1^. GrO is obtained by oxidizing process of graphite, and it’s a precursor to graphene. It is formed by sp ^2^-hybridized carbon atoms arranged in a hexagonal lattice which act as a barrier to gas molecules when oriented perpendicular to the graphene plane^2,3^. It has a unique feature such as high surface area and several oxygen-containing functional groups distributed on both edges and basal plane^4,5^. Graphene oxide (GrO) exhibits several notable electronic features due to its unique structure and composition. It has adjustable electronic features, including a tunable work function ranging from 3.7 to 5.1 eV, which allows it to be used as a hole or electron transport material in various electronic devices^6^. Despite the presence of oxygen functional groups, GrO maintains high conductivity, making it suitable for applications in electronic devices and sensors^7,8^. GrO possesses a unique electronic band structure with small band gaps, which contributes to its fast charge mobility and excellent electrical transport properties^8^. The surface of GrO is rich in oxygen-containing functional groups like epoxides, carboxylic acids, and hydroxyl groups. This enables simple covalent binding of biomolecules, significantly boosting its utility in biosensors and electronic applications^9,10^. The chemical stability, and the electronic properties of GrO makes it a robust material for various electronic applications, including sensors and energy storage^7^. GrO can undergo further chemical modifications to lower its work function, facilitating electron injection and extraction in organic electronic devices^11^.

GrO could be further enhanced with Iron oxide (FeO), a combination which has been studied due to the exceptional characteristics of both materials. For supercapacitors and sensors application GrO/FeO shows large surface area beside the high conductivity as reported earlier^12^. GrO/FeO composite is the dedicated for many practical applications, as the graphene sheets show a tendency to avoid the aggregation process of FeO^13,14^ which in turn enhance its application. Additionally, the composite benefits from a large surface area and high conductivity. The GrO/FeO composite can improve oxygen evolution reaction, this could be conducted through interactions with graphene functional groups especially those contained oxygen, this provides electrical conductivity beside enhancing the mechanical strength^15^.

Beyond iron oxide (FeO), other doping and/or treatment methods for GrO, such as interaction with nitrogen, can significantly enhance both its reactivity and stability^16,17^. Nitrogen serves as an effective dopant because its electron pair increases catalyst conductivity. It’s been reported that N-doped carbon materials provide functional groups, like hydroxyl groups, which boost reactivity^18^. Generally, nitrogen-doped carbon materials show an increase in active sites without altering the electronic properties of the carbon. Nitrogen also helps maintain the skeletal structures of carbon-based materials since its atomic radius is similar to that of carbon^19,20^. This paves the way for the practical fabrication of nitrogen-doped carbon nanomaterials with pyridinic N and graphitic N^21^, making these composites ideal for use as catalysts and sensing materials^17,21,22^.

GrO can also be doped with sulfur in various forms, including thiophene and sulfur oxide. Studies indicate that sulfur atoms can be absorbed on graphene surfaces, substitute carbon atoms at the edges, or connect graphene sheets by forming sulfur clusters^23^. The resulting GrO/sulfur oxide (GrO/SO) materials boast enhanced electronic properties and an abundance of active sites for charge storage, enabling their potential application in energy storage and electrocatalysis^24^.

As far as the studied GrO doped with metal oxides such as FeO or nonmetal oxides such as NO and/or SO it was found that the electronic properties are enhanced. One of the promising tools for elucidating electronic properties is the computational methods such as density functional theory DFT^25^.

DFT is an important tool to explore the total dipole moment, HOMO/LUMO band gap energy, electrostatic potential, Infrared spectra, Raman spectra and thermochemical parameters^26–30^. Throughout DFT one also can describe the Fukui functions as an indicators or reactivity descriptors^31^. Also, one can consult the DFT to check and describe the stability of the studied compounds through the theory of atoms in molecules QTAIM^32–34^. For GrO/FeO composite DFT is reported as an essential tool for studying the interaction mechanisms beside its role for validating the experimental findings^35^. DFT has been conducted to explore the GrO/FeO composite. DFT indicated that, GrO contain functional groups which enhanced the binding energy with FeO, by forming interfacial C − O covalent bonds^36^. DFT also investigated the electronic and magnetic properties of FeO. Modified DFT calculations could provide good agreement with experimental observations^37^. Non metal oxide such as NO is proven to interact strongly with GrO as indicated earlier by DFT^38^. The mechanism of N interaction with GrO could be also described with DFT^39^. It was indicated that the NO is providing additional active sites for GrO which in turn enhances its reactivity and functionality. GrO doped with S was subjected for DFT indicating that, DFT is providing detailed insights into electronic properties, stability, and interactions^40,41^. DFT could optimize geometry and provide several parameters such as lattice stability and charge transfer at GrO/SO interfaces.

DFT was conducted upon six designed π-conjugated chromogens, to incorporate a heteroaromatic carbazole framework as a robust electron donor, cyano and nitro groups as electron acceptors, and a phenyl linker/auxiliary donor. The electronic distribution was assessed in their frontier molecular orbitals, including optimized geometries, energy levels, and molecular mapping^42^. Time-Dependent Density Functional Theory TD-DFT calculations were conducted to evaluate the electronic characteristics of simple carbazole-derived organic dyes to evaluate their possible optoelectronic applications^43^.

Based on these considerations, the present work is applying DFT to study Gr, GrO and GrO interacted with FeO, NO and SO. Upon optimization of the studied structures, the total dipole moments, HOMO/LUMO energy gaps, global reactivity descriptors, density of states, molecular electrostatic potential, Quantum Theory of Atoms in Molecules topologies QTAIM, and noncovalent interaction patterns were calculated with B3LYP/LANL2MB level of theory.

## Materials and methods

### Building model molecules

**Fig. 1:**
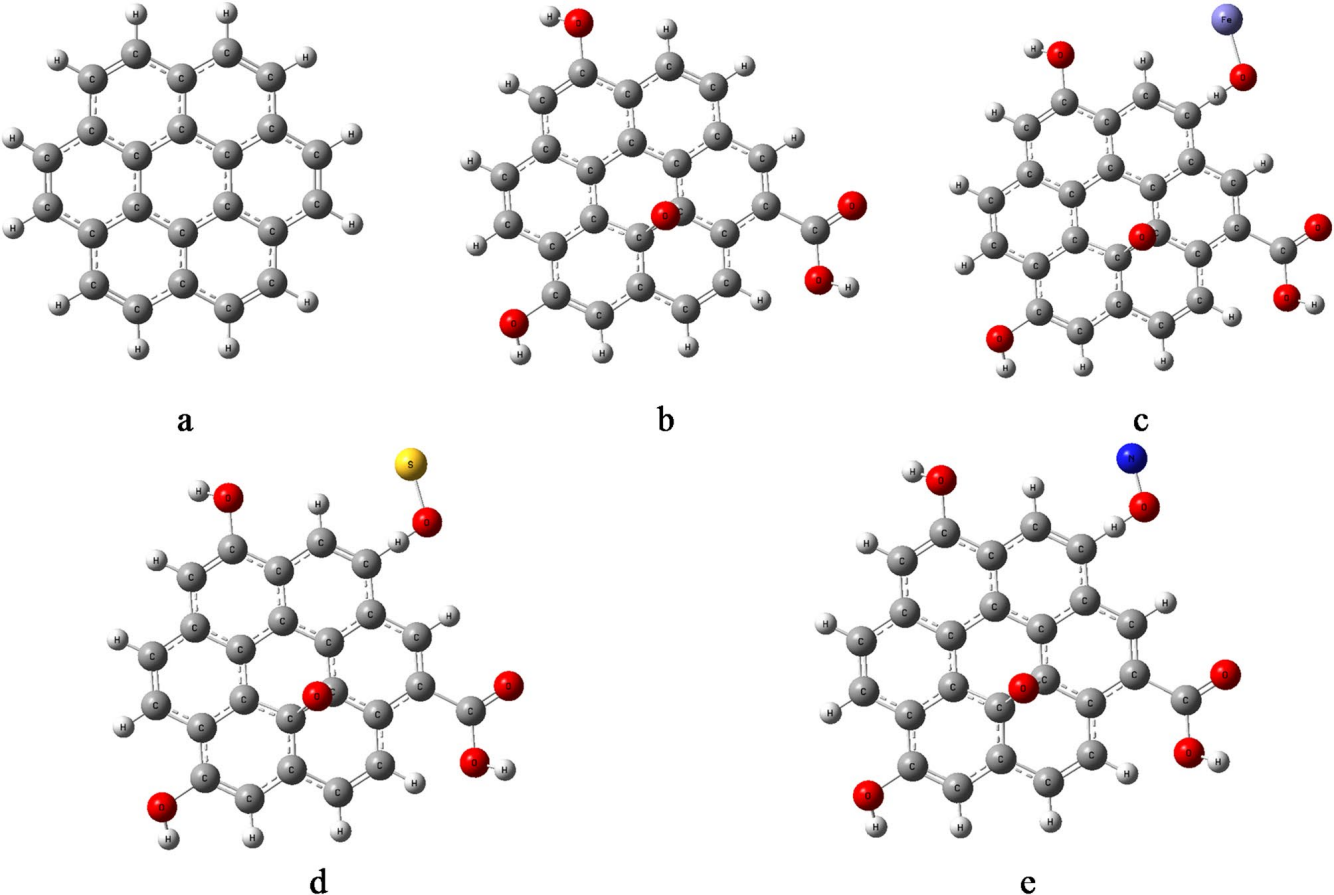
The studied model structures whereas; a- G, b- GrO, c- GrO/FeO, d- GrO/SO, and e- GrO/NO.

The first step in this computational work is to describe the structure of the studied model molecules. Model structures for graphene, graphene oxide, and its decorated forms were optimized using DFT calculations at the B3LYP/LANL2MB level of theory. The pristine graphene model (Fig. 1a) was oxidized to obtain the graphene oxide (GrO) model by introducing epoxy and carboxyl functional groups, as shown in Fig. 1b. The GrO model was then decorated with FeO containing Fe⁺ to form GrO/FeO (Fig. 1c). Additionally, GrO was decorated with SO and NO species carrying charges of S⁻¹ and N⁻¹, respectively, to form GrO/SO and GrO/NO, illustrated in Fig. 1d and e.

### Calculation details

The second step is to describe the computational efforts which is conducted upon the designed model molecules. quantum calculations for model structures were conducted at Molecular modeling and Spectroscopy Laboratory, Centre of Excellence for Advanced Science, National Research Centre, Egypt, using Gaussian 09 software^44^ at B3LYP^45–47^ functional and LANL2MB basis set. Several important parameters at this same theoretical level were calculated, including total dipole moment (TDM), HOMO/LUMO band gap energy (ΔE), and density of states (DOS) and projected density of states (PDOS). Also, molecular electrostatic potential (MESP) was mapped. From the HOMO and LUMO energy values, global reactivity descriptors were derived, such as ionization potential (I), electronic affinity (A), chemical potential (µ), chemical hardness (η), absolute chemical softness (S), and electrophilicity index (ω), using the Eqs. (1–6)^32^:

1 $${\text{I }}={\text{ }} - {{\text{E}}_{{\text{HOMO}}}}$$

2 $${\text{A}}\,=\, - \,{{\text{E}}_{{\text{LUMO}}}}$$

3 $$\mu ={\text{ }} - \left( {{\text{I}}\,+\,{\text{A}}} \right)/{\text{2}}$$

4 $$\eta =\left( {{\text{I}} - {\text{A}}} \right)/{\text{2}}$$

5 $${\text{S}}\,=\,{\text{1}}/\eta$$

6 $$\omega \, = \,\mu ^{{\text{2}}} /{\text{2}}\eta$$

To ensure the stability of the studied models, Quantum Theory of Atoms in Molecules (QTAIM) calculations were carried away. These calculations were performed using Multiwfn^48^ and Visual Molecular Dynamics (VMD) software^49^.

## Results and discussion

### Total density of states (TDM) and HOMO/LUMO energy gap

Some important physical parameters to evaluate the changes in electronic properties for the studied structures is to be investigated. Total dipole moments and HOMO–LUMO energy gaps were computed at the B3LYP/LANL2MB level of theory for all graphene-based structures. The results presented in Table 1 that shows how oxidation and subsequent decoration dramatically alter both polarity and electronic excitation energy. Pristine graphene (Gr) exhibits a zero total dipole moment (TDM = 0.000 Debye) due to its perfect symmetry, and a relatively wide HOMO–LUMO gap of 4.4831 eV. Graphene oxide (GrO) breaks the symmetry by introducing epoxy and carboxyl groups. This yields a modest dipole moment of 3.0568 Debye and reduces the energy gap to 3.2256 eV. Decoration of GrO with Fe, SO, or NO further perturbs both TDM and ΔE. For GrO/Fe, TDM leaps to 14.2613 Debye, reflecting strong charge separation around the FeO moiety. Correspondingly, ΔE drops to 1.6245 eV. This significant narrowing of the gap suggests electrons can be excited much more readily—an effect attributable to Fe’s unfilled d-orbitals introducing new frontier orbitals at lower energies. For GrO/SO, TDM reaches its maximum at 20.3783 Debye, indicating the SO fragment promotes charge separation for the composite. The HOMO–LUMO gap shrinks to a mere 0.5761 eV, the smallest among all systems. Such a low ΔE implies near-semimetallic behavior and suggests very high electrical conductivity or sensitivity toward moieties (e.g., gas adsorption). For GrO/NO, TDM is 2.9034 Debye, slightly lower than pure GrO, while ΔE is 2.4115 eV. Here, NO’s influence on dipolarity is milder compared to FeO or SO, and its frontier orbitals reside closer to those of the GrO backbone, producing a gap that sits between the extremes of GrO and GrO/SO. These trends underscore that the choice of decorating species directly controls both charge separation and the ease of electron excitation, it could be applied for tuning sensing performance or catalytic activity. As listed also in Table 1, the experimental HOMO/LUMO energy gap (ΔE) in eV f for GrO decorated with FeO, SO and NO show comparable results with those obtained with molecular modeling at B3LYP/LANL2MB level.

**Table 1:** Calculated total dipole moment (TDM) in Debye and HOMO/LUMO energy gap (ΔE) in eV for the studied systems.

Structures	TDM (Debye)	ΔE (eV)	Exp., ΔE (eV)
Gr	0.000	4.4831	
GrO	3.0568	3.2256	
GrO/Fe	14.2613	1.6245	2.0–2.4^50^
GrO/SO	20.3783	0.5761	2.3–2.8^51^
GrO/NO	2.9034	2.4115	1.5–2.5^52^

### Frontier molecular orbitals (FMOs)

**Fig. 2:**
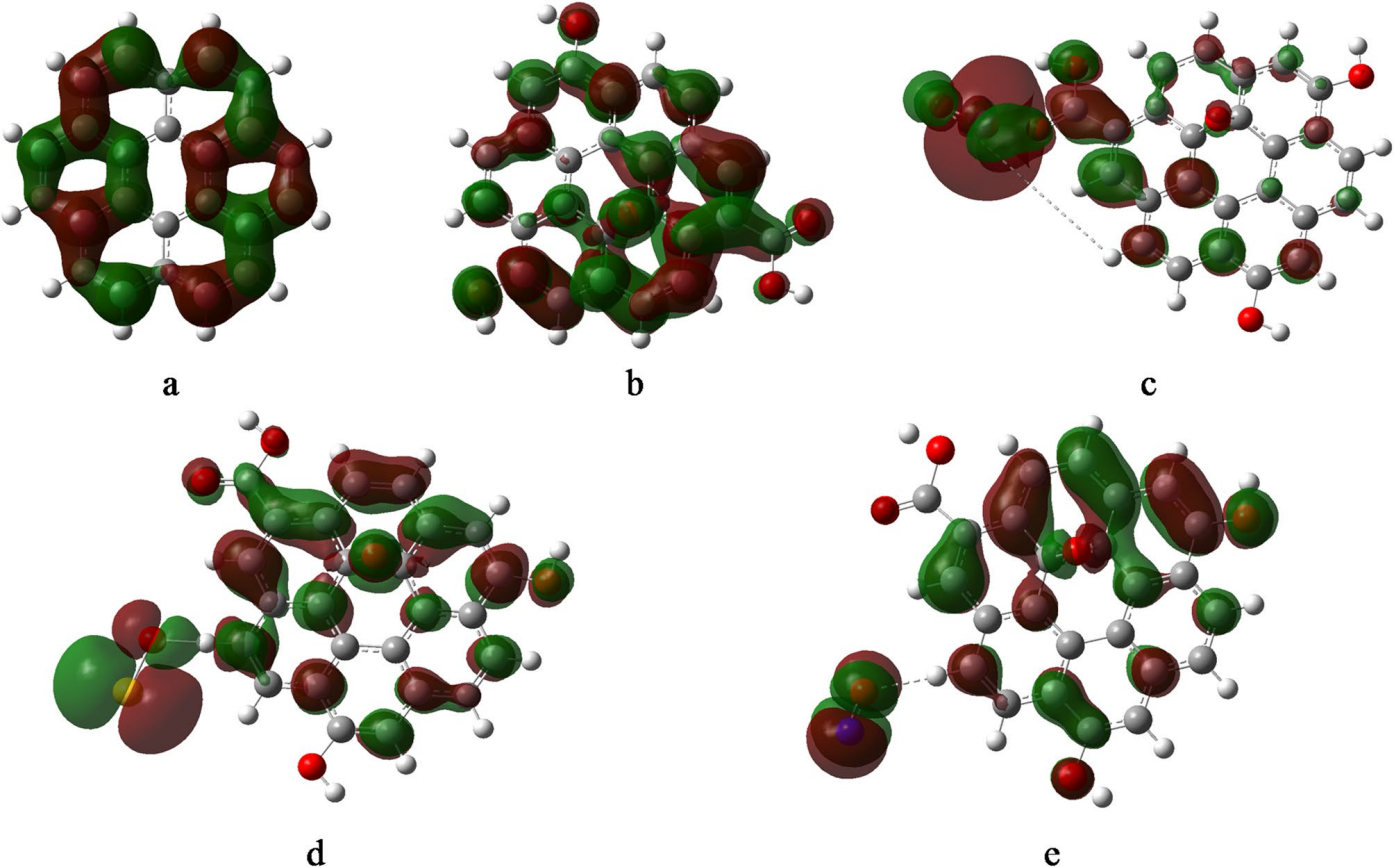
Frontier HOMO and LUMO calculated at B3LYP/LANL2MB level for the studied systems whereas; a- Gr, b- GrO, c- GrO/FeO, d- GrO/SO, and e- GrO/NO.

HOMO–LUMO frontier orbitals (FMO) were calculated for graphene oxide (GrO) and its decorated derivatives at the B3LYP/LANL2MB level of theory and are presented in Fig. 2. Frontier molecular (FMOs) orbitals for Gr shows homogenous distribution over its π-framework, while GrO structure displays moderately delocalized HOMO and LUMO orbitals around the oxygenated regions. Upon decoration, the FMOs exhibit significant redistribution, reflecting localized electronic interactions with the attached species. For GrO/FeO, the HOMO is predominantly localized around the FeO moiety, indicating potential electron-donating behavior centered on the Fe site, while the LUMO remains broadly distributed. In GrO/SO, the HOMO is concentrated on the SO group, whereas the LUMO extends across the GrO surface, suggesting a possible charge-transfer pathway from SO to the GrO surface. Conversely, in GrO/NO, the LUMO is highly localized on the NO group, with the HOMO delocalized over the GrO framework, implying an electron-accepting role for the NO group. These FMO patterns highlight the distinct electronic modifications induced by each decorating species and their influence on charge distribution within the composite systems.

### Density of States (DOS) and projected density of States (PDOS)

**Fig. 3:**
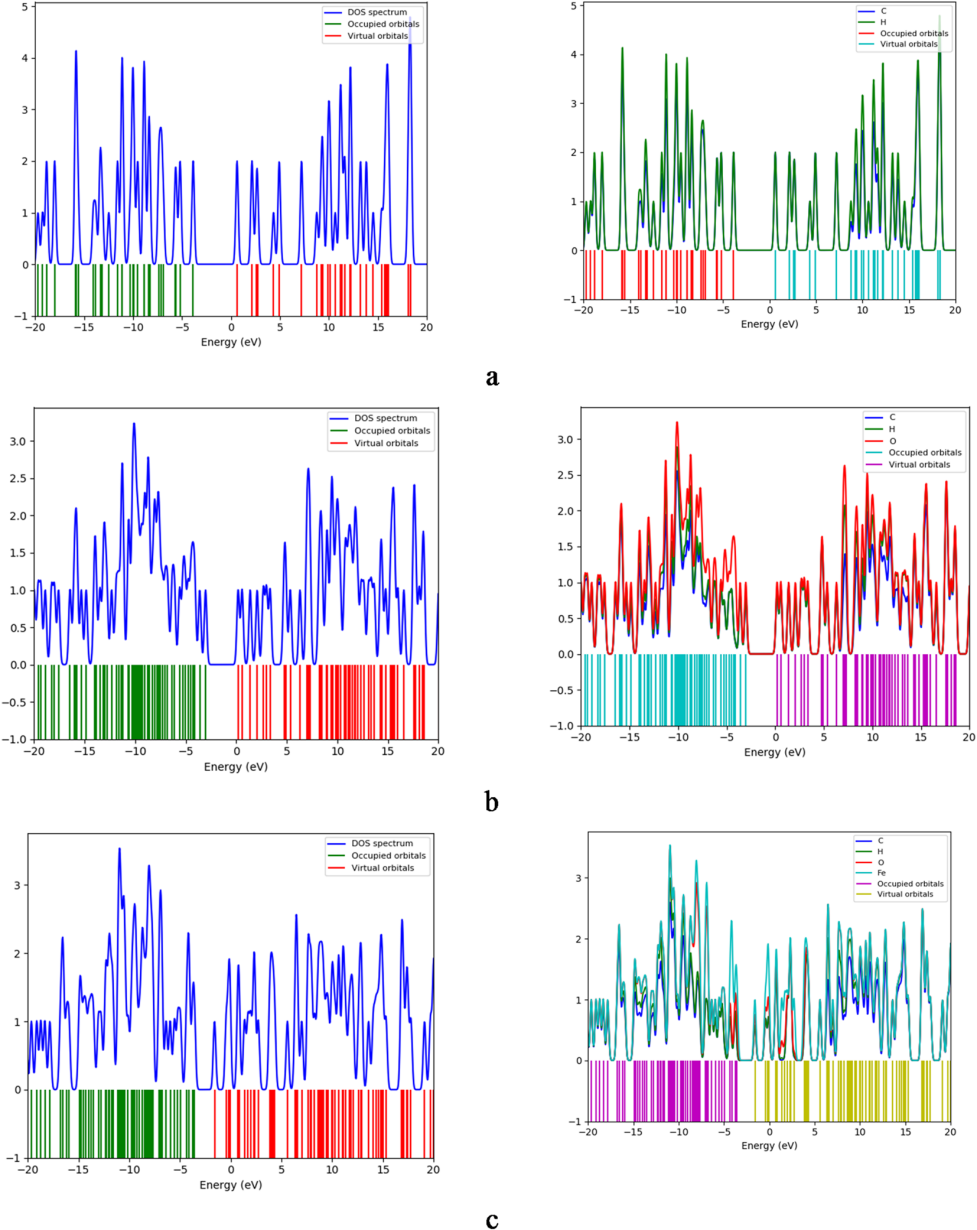

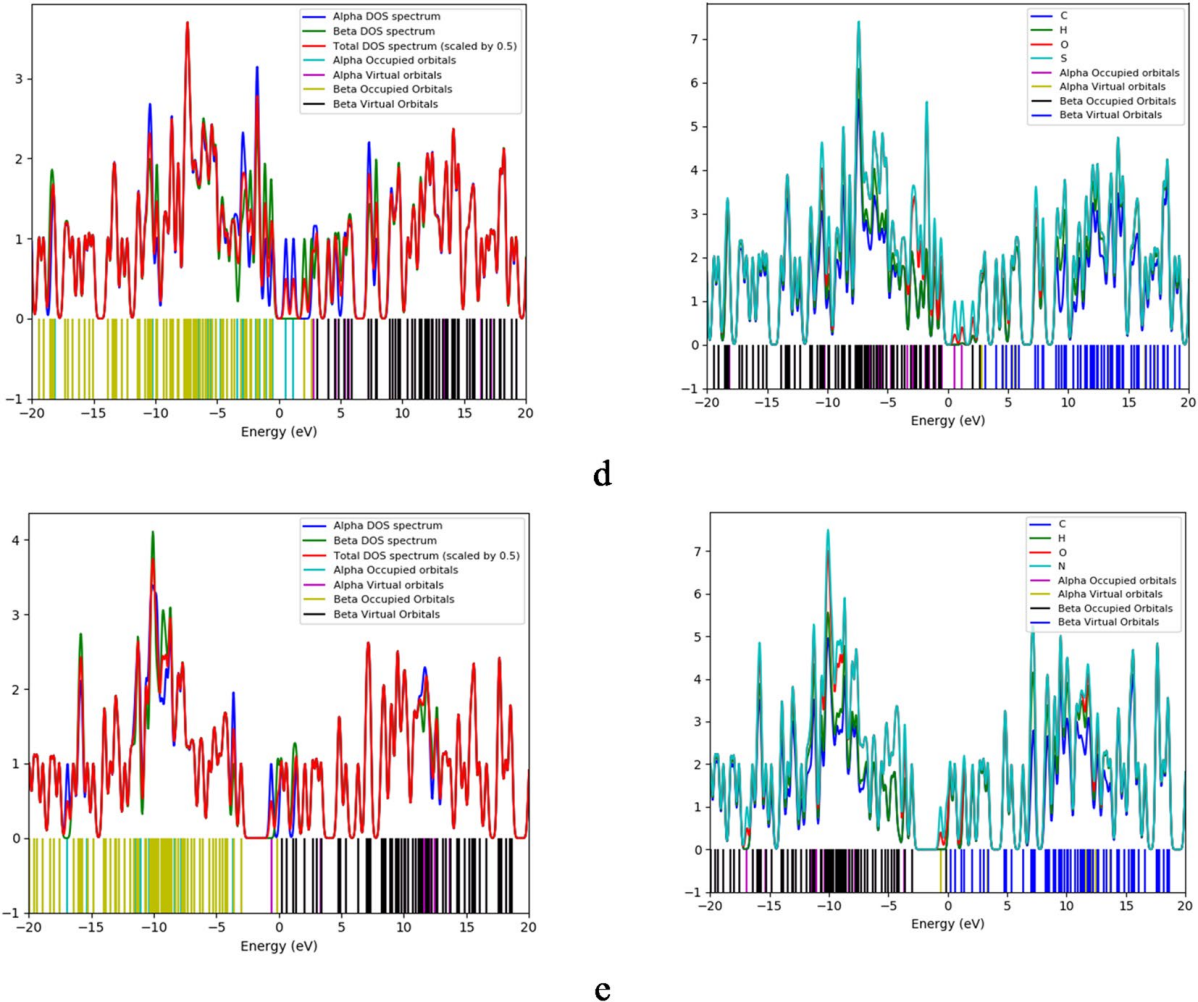
Calculated DOS and PDOS for the studied systems whereas; a- Gr, b- GrO, c- GrO/FeO, d- GrO/SO, and e- GrO/NO.

The total density of states (DOS) for all studied structures was calculated at the B3LYP/LANL2MB level of theory and is shown in Fig. 3. DOS plots represent the number of electronic states available at each energy level where green lines indicate occupied states, while red lines indicate unoccupied (virtual) states. The bandgap is the energy region between the highest occupied state and the lowest unoccupied state. Results show that the bandgap of graphene oxide (GrO) is smaller than that of pristine graphene (Gr) and this gap narrows further for the decorated systems. In particular, GrO/SO exhibits the smallest gap, with a pronounced increase in unoccupied states appearing near the Fermi level. This shift of virtual orbitals toward the Fermi energy facilitates enhanced charge transfer in the decorated composites. Projected density of states (PDOS) plots decomposes the total DOS into contributions from individual atomic orbitals. In Fig. 3, PDOS analysis shows that Fe, S, and N atoms contribute most significantly to both occupied and unoccupied states in their respective GrO/FeO, GrO/SO, and GrO/NO structures. Oxygen atoms also make notable contributions, but to a lesser extent than Fe, S, or N.

These PDOS features confirm that the decorating atoms introduce new electronic states near the Fermi level, thereby modulating the band structure and improving charge-transfer characteristics.

### Molecular electrostatic potential (MESP)

**Fig. 4:**
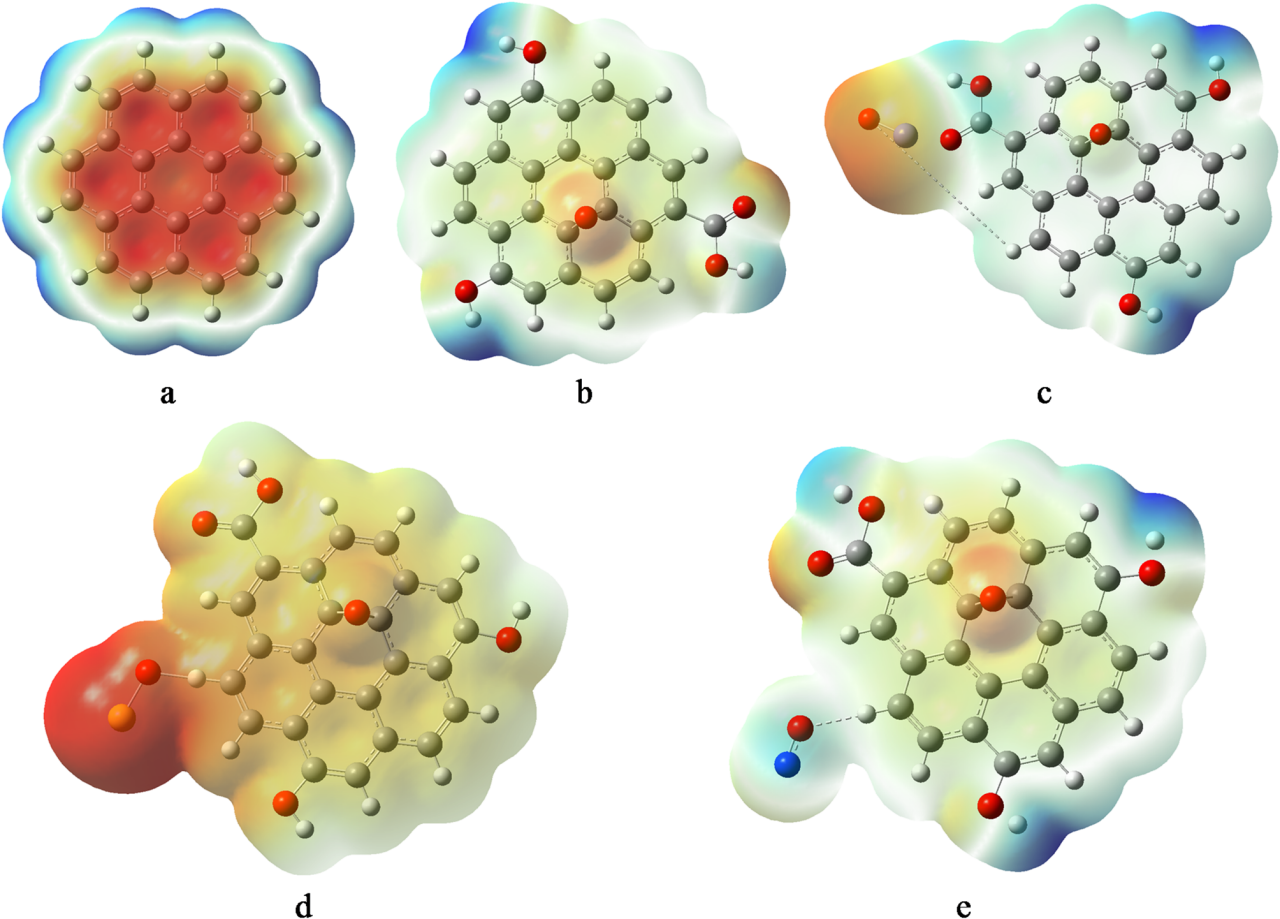
Calculated MESP for the studied systems whereas; a- Gr, b- GrO, c- GrO/FeO, d- GrO/SO, and e- GrO/NO.

The molecular electrostatic potential (MESP) was calculated using the B3LYP/LANL2MB level of theory. MESP maps provide insight into the reactive regions of molecules by visually depicting the distribution of electrostatic potential across the molecular surface. These maps use a color gradient from red to blue, where red denotes regions of negative potential (electron-rich), blue represents positive potential (electron-deficient), and green indicates areas of near-neutral charge^53^. Figure 4 presents the MESP distributions for the graphene-based structures. In pristine graphene (Gr, Fig. 4a), the surface exhibits a predominantly negative potential, reflecting its uniform delocalized π-electron cloud. Upon oxidation to form graphene oxide (GrO), the electrostatic potential becomes more varied, blue regions appear near hydroxyl groups, indicating localized positive potential, while red regions are observed on epoxy oxygen atoms, highlighting electron-rich sites. Following decoration, the electrostatic profiles change significantly. In GrO/FeO, a pronounced red region is observed around the FeO unit, indicating strong electron density accumulation. GrO/SO displays a highly active surface, with intense red coloration around the SO group and more moderate yellowish tones across the remaining structure, suggesting enhanced surface reactivity. The GrO/NO system exhibits a distribution similar to GrO but shows a notable positive potential localized around the NO group, in line with the electron-withdrawing nature of NO. These MESP variations suggest significant changes in chemical reactivity and interaction potential upon functionalization, particularly at the decoration sites.

### Global reactivity descriptors

To further explore the chemical reactivity and stability of the studied graphene-based structures, several global reactivity descriptors were calculated, including ionization potential (I), electron affinity (A), chemical potential (µ), global hardness (η), global softness (S), and electrophilicity index (ω). Table 2 presented the calculated global reactivity descriptors at B3LYP/LANL2MB for the studied structures. Data in Table 2 provide valuable parameters which indicated the tendency of a molecule to donate or accept electrons, as well as its overall reactivity and stability. For pristine Gr, the calculated ionization potential was 0.6144 eV and the electron affinity was − 3.8686 eV, a chemical potential of 3.8686 eV and a low global hardness of − 0.6144 eV. These values indicate high electronic stability but low chemical reactivity, consistent with the known inert nature of pristine graphene. For GrO, the ionization potential decreased to 0.1948 eV, while the electron affinity increased to − 3.0308 eV. This shift led to a slightly lower chemical potential and softness, indicating a modest increase in reactivity due to the presence of oxygen-containing functional groups. More pronounced changes were observed upon further decoration. The GrO/FeO structure exhibited a significantly higher ionization potential (2.8101 eV) and positive electron affinity (1.1856 eV), resulting in a negative chemical potential and increased global softness. This suggests enhanced electrophilicity, high reactivity and a higher tendency to participate in charge-transfer interactions. Similarly, GrO/SO showed comparable behavior, with a chemical potential of − 2.0730 eV, the highest global softness (2.3610 eV^-1^) and an even lower electrophilicity index (0.2880 eV) and even indicating its potential as a reactive surface, particularly in oxidative or redox environments. In contrast, GrO/NO displayed a unique profile, with a negative ionization potential (–0.6071 eV) and a more negative electron affinity (–3.0186 eV). These values led to a positive chemical potential, implying distinct electronic behavior compared to the other systems. Although its global softness and electrophilicity are lower than those of GrO/FeO and GrO/SO, the GrO/NO structure still demonstrates a significant modification in reactivity compared to pristine and oxidized graphene. Overall, the introduction of functional groups and metal oxides significantly alters the global reactivity descriptors of the graphene framework, enhancing the tunability of its electronic properties for targeted applications such as sensing, catalysis, or charge transport. Collecting these data one can conclude that, GrO is slightly more reactive than pristine graphene. Doping GrO is significantly altering electronic properties. GrO/SO has the highest softness (S), indicating it’s the most chemically reactive and potentially best suited for catalytic or sensing applications. While GrO/FeO shows high ionization potential and hardness, suggesting greater stability and lower reactivity. This is suitable for electronic or energy storage applications. Finally, the GrO/NO has negatived I and A, which is physically non-intuitive, this may reflect a computational artifact or an unusual electronic configuration that needs further validation.

**Table 2:** Calculated global reactivity descriptors at B3LYP/LANL2MB for the studied structures.

Structures	I (eV)	A (eV)	µ (eV)	η (eV)	S (eV)^−1^	ω (eV)
Gr	0.6144	-3.8686	3.8686	-0.6144	-1.6271	2.2415
GrO	0.1948	-3.0308	3.0308	-0.1948	-1.4180	1.6128
GrO/FeO	2.8101	1.1856	-1.1856	-2.8101	1.9979	0.8123
GrO/SO	2.6490	2.0730	-2.0730	-2.6490	2.3610	0.2880
GrO/NO	-0.6071	-3.0186	3.0186	0.6071	-1.8128	1.2057

### Quantum theory of atoms in molecules analysis

**Fig. 5:**
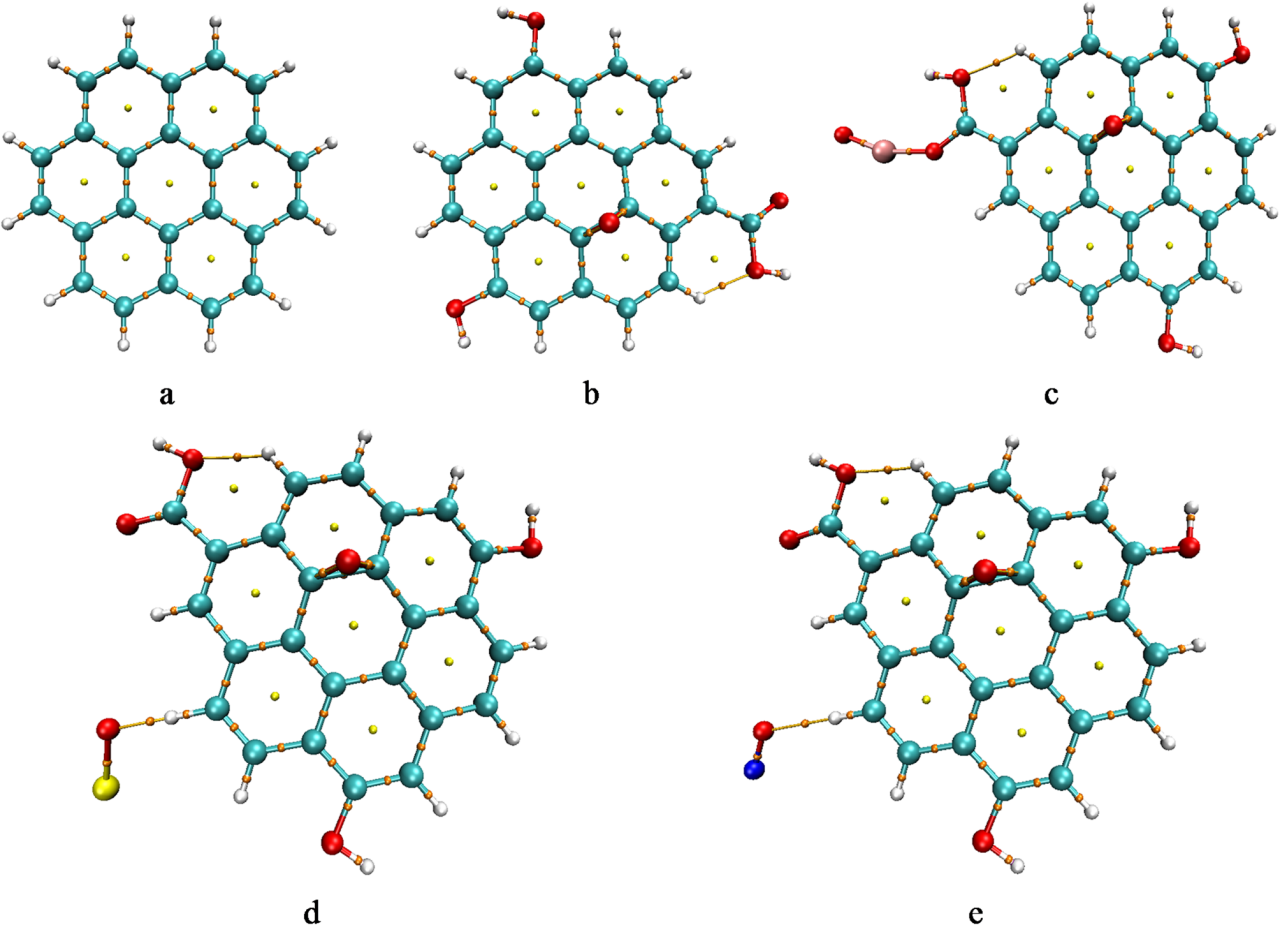
Calculated QTAIM topology for the studied systems whereas; a- G, b- GrO, c- GrO/FeO, d- GrO/SO, and e- GrO/NO.

QTAIM analysis was employed to investigate the nature of interactions between the graphene oxide framework and the decorating species (FeO, SO, and NO) by examining key topological parameters such as the electron density ρ(r), the Laplacian of the electron density ∇²ρ(r), and the total energy density H(r) at the bond critical points (BCPs)^33–35^. These values provide insight into whether the interactions are covalent, ionic, or non-covalent in nature, such as hydrogen bonding or van der Waals forces. The QTAIM topology for the studied systems calculated at the same level of theory presented in Fig. 5. For the GrO/FeO system, the interaction between graphene oxide and FeO was characterized by a relatively high electron density at the BCP (ρ(r) = 0.1403 a.u.), a positive Laplacian (∇²ρ(r) = 0.9719 a.u.), and a negative energy density (H(r) = − 0.0391 a.u.). This combination of a high electron density and negative H(r) typically signifies a covalent interaction with partial ionic character, confirming a strong and localized bond between FeO and the graphene oxide surface. In contrast, both GrO/SO and GrO/NO complexes showed features consistent with hydrogen bonding. For GrO/SO, the electron density was ρ(r) = 0.0684 a.u., with a positive Laplacian (∇²ρ(r) = 0.2293 a.u.) and a small positive energy density (H(r) = 0.0022 a.u.), indicative of a closed-shell interaction. Similarly, the GrO/NO structure exhibited even lower electron density at the BCP (ρ(r) = 0.0136 a.u.), with ∇²ρ(r) = 0.0725 a.u. and H(r) = 0.0031 a.u., further confirming the presence of a weak, non-covalent hydrogen bond. Such bonding differences are expected to significantly influence the electronic distribution and chemical reactivity of the respective composite materials.

### Non covalent interactions (NCI) analysis

**Fig. 6:**
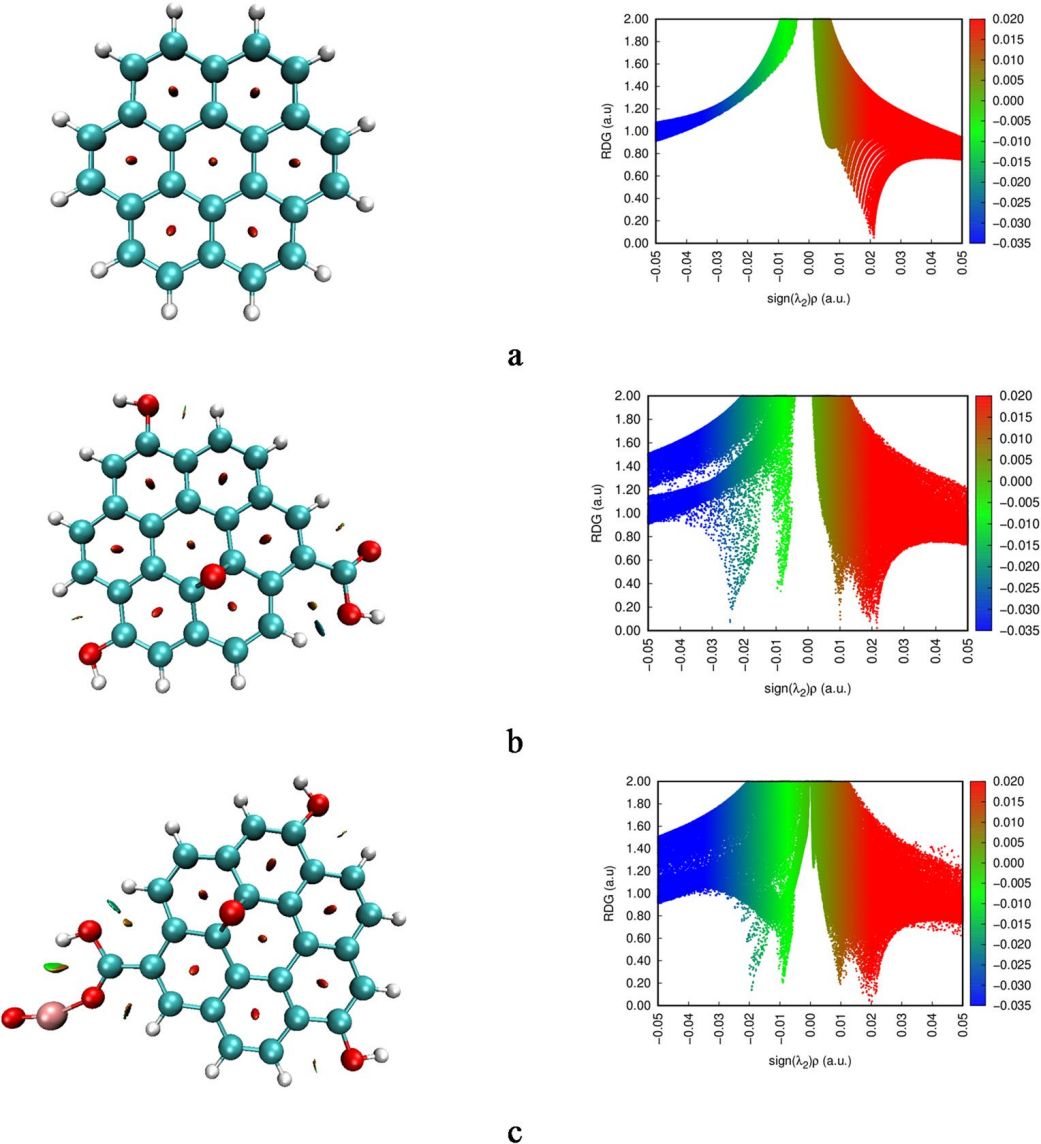

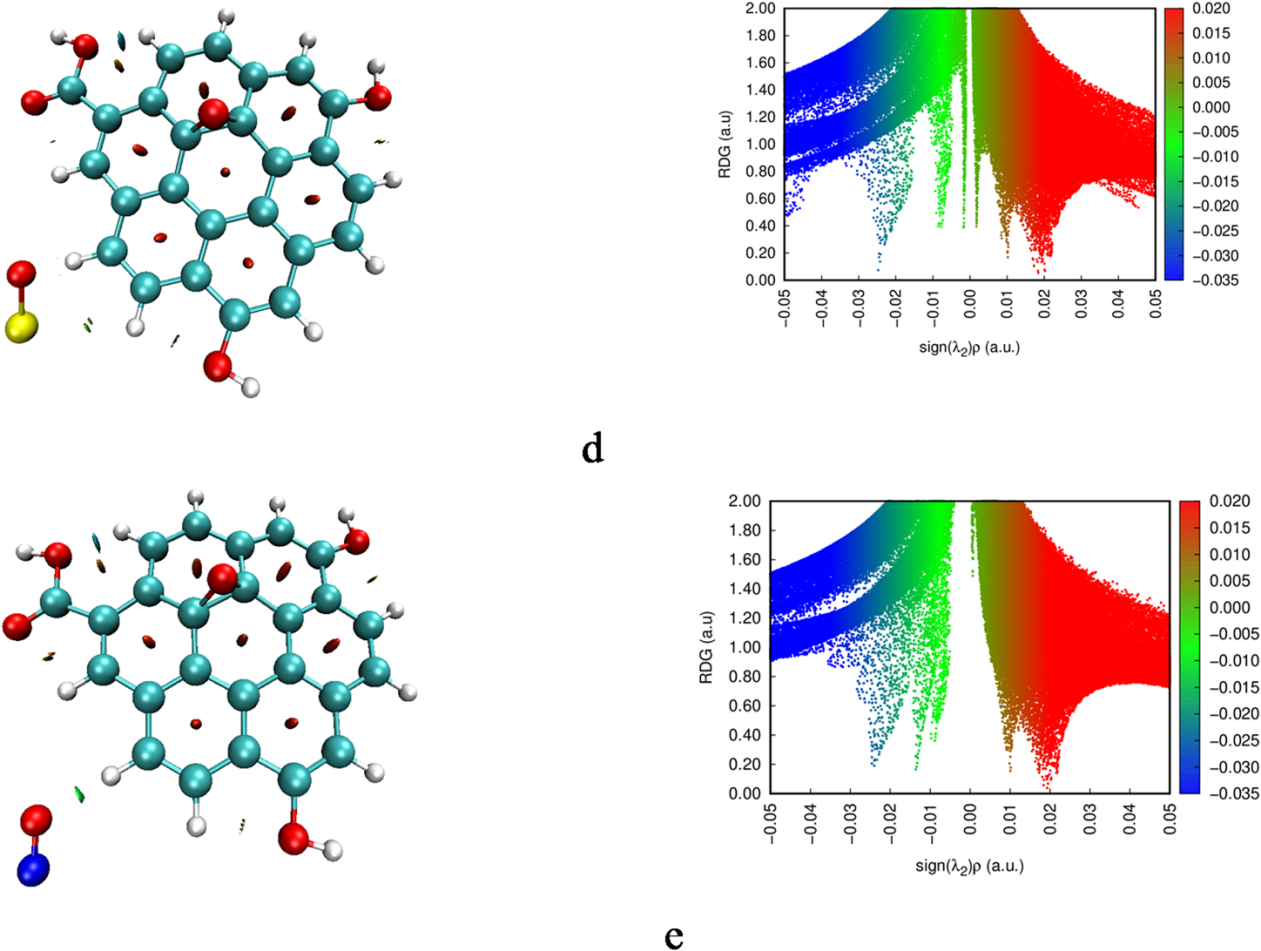
Calculated NCI and RDG scatter plots for the studied systems whereas; a- Gr, b- GrO, c- GrO/FeO, d- GrO/SO and e- GrO/NO.

To further elucidate weak interactions in the studied composites, NCI analysis combined with Reduced Density Gradient (RDG) mapping was performed at the B3LYP/LANL2MB level of theory. These techniques visualize noncovalent forces through isosurfaces colored where blue regions indicate strong attractive interactions (hydrogen bonds); green denotes van der Waals (vdW) interactions, and red represent steric repulsion^54^. The resulting NCI isosurfaces and corresponding RDG plots are presented in Fig. 6. In pristine graphene (Gr), the NCI isosurfaces reveals red isospheres in between carbon rings, reflecting steric repulsion within the hexagonal network. Neither blue or green isosurfaces nor spikes in RDG appear, confirming the absence of significant hydrogen bonding or vdWs interactions. Upon oxidation (GrO), the steric repulsion pattern between carbon rings remains essentially unchanged. However, additional blue isosurfaces emerge around the hydrogen atoms of hydroxyl and carboxyl groups interacting with nearby oxygen atoms, as corroborated by RDG spikes. This indicates that GrO exhibits both intramolecular steric repulsion and localized hydrogen bonding due to its functional groups. In the GrO/FeO composite, RDG mapping shows green isosurfaces in the region between FeO and GrO site of interaction, indicating vdW interactions dominate this interface. The absence of any neither blue isospheres nor blue spikes in RDG confirms that no significant hydrogen bonding occurs between FeO and GrO. For GrO/SO, the NCI isosurfaces similarly feature green isospheres between the SO moiety and adjacent GrO atoms, indicating enhanced vdW contacts compared to GrO alone. This means SO is stabilized primarily by vdW forces. Finally, in GrO/NO, distinct green isosurfaces appear between NO and the GrO sheet, marking vdW interactions rather than hydrogen bonds. Although NO can sometimes engage in weak H···N contacts, no blue regions are observed. The RDG scatter shows a green-colored spike aligning with the presence of weak vdWs, and broader blue spikes confirming hydrogen bonding that has been found in QTAIM analysis.

## Conclusion

Decorating graphene oxide with FeO, SO, or NO dramatically alters its polarity, band gap, and chemical reactivity, thereby offering a versatile route to engineer GrO-based materials for electrode and sensing applications. Oxidation alone generates a moderate dipole moment and narrows the HOMO–LUMO gap compared to pristine graphene. Subsequent decoration with FeO or SO further amplifies charge separation (TDM = 14.26 Debye and 20.38 Debye, respectively) and collapses the gap (ΔE = 1.625 eV for FeO, 0.576 eV for SO), while NO decoration produces intermediate changes.

The experimental ΔE for the decorated GrO/(FeO, SO and NO) showed comparable results with those calculated, which is an experimental validation for the studied B3LYP/LANL2MB model.

Global reactivity descriptors reveal that GrO/FeO and GrO/SO behave as soft, strongly electrophilic species, ideal for facile charge transfer and redox activity, whereas GrO/NO remains moderately reactive. DOS and PDOS confirm that Fe, S, and N-derived orbitals contribute prominently near the Fermi level, boosting electronic conductivity. MESP mappings pinpoint active sites on each decorated model with GrO/SO showing high reactive surface, and QTAIM analysis clearly demonstrates that FeO forms a covalent Fe-O linkage to GrO, whereas SO and NO engage via hydrogen bonds. Finally, NCI visualization underscores the dominance of van der Waals interactions at all decoration interfaces, with no new covalent contacts beyond the Fe-O bond. Collectively, these findings indicate that GrO/SO combines exceptionally high dipolarity, minimal bandgap, and abundant reactive surface, rendering it particularly well suited for next-generation electrode materials and gas sensors. Future work will explore explicit interactions with target analytes and extend these modifications to experimental validations in device contexts. Based on the provided data, we can draw the following conclusions:

Graphene oxide (GrO) is slightly more reactive than pristine graphene.

Doping significantly alters the electronic properties of GrO.

GrO/SO has the highest softness (S), making it the most chemically reactive derivative. This high reactivity suggests it’s a good candidate for catalytic or sensing applications.

GrO/FeO has a high ionization potential and hardness. This indicates it’s more stable and less reactive, making it suitable for electronic or energy storage applications.

GrO/NO shows negative ionization potential (I) and electron affinity (A). This is not physically intuitive and likely represents a computational artifact or an unusual electronic configuration that requires further investigation.

## Data Availability

The data supporting the findings of this study can be obtained from the corresponding author upon request, subject to reasonable conditions.
